# Bacteriophage therapy against multidrug resistant bacterial infections demonstrates clinical advances and engineering innovations between 2020–2026

**DOI:** 10.3389/fmicb.2026.1865548

**Published:** 2026-06-05

**Authors:** Ahmed J. Alzahrani

**Affiliations:** College of Medicine, Al-Imam Mohammad Ibn Saud Islamic University, Riyadh, Saudi Arabia

**Keywords:** antimicrobial resistance, bacteriophage therapy, combination therapy, CRISPR-Cas systems, multi-drug resistance, phage engineering

## Abstract

**Purpose:**

The global crisis of antimicrobial resistance has reached critical levels, with multi-drug resistant (MDR) bacterial pathogens threatening to render conventional antibiotics ineffective. This mini review synthesizes recent evidence from 2020 to 2026 on bacteriophage therapy against MDR bacteria, examining clinical applications, engineering advances, mechanistic insights, and emerging technologies.

**Methods:**

A comprehensive literature search was conducted across Embase, Scopus, and Cochrane Library databases, supplemented by PubMed, Google Scholar, and ArXiv searches, focusing on phage therapy for MDR bacterial infections published between 2020 and 2026.

**Results:**

Recent developments include expanded clinical experience through specialized phage centers, sophisticated genetic engineering techniques including CRISPR-based systems, successful compassionate-use programs, and innovative combination therapies with antibiotics. Clinical reports demonstrate safety and preliminary efficacy signals in selected refractory infections, though randomized controlled data remain limited. Engineering advances have produced phage-delivered CRISPR antimicrobials, hybrid delivery platforms, and synthetic phage particles that expand therapeutic capabilities.

**Conclusion:**

While challenges remain in regulatory standardization, scalable manufacturing, and resistance management, the field has demonstrated significant progress toward clinical translation. The convergence of synthetic biology, personalized medicine approaches, and growing clinical evidence positions phage therapy as a viable complementary strategy in the fight against MDR bacterial infections.

## 1 Introduction

### 1.1 The Antibiotic resistance crisis

The accelerating global burden of antimicrobial resistance represents one of the most pressing challenges in modern medicine, fundamentally threatening our ability to treat common bacterial infections. The development of new antibiotic classes has dramatically slowed over recent decades, while bacterial pathogens have continued to evolve resistance mechanisms at an alarming rate. Carbapenem-resistant Gram-negative bacteria, methicillin-resistant *Staphylococcus aureus* (MRSA), and other priority pathogens designated by the World Health Organization now cause infections with limited or no effective treatment options (Gorski et al., 2020; Hatfull et al., 2022). This crisis has created an urgent need for alternative therapeutic approaches that can circumvent traditional resistance mechanisms.

### 1.2 Bacteriophages as precision antibacterials

Bacteriophages, viruses that specifically infect and kill bacteria, offer unique therapeutic advantages that distinguish them from conventional antibiotics. Their high host specificity allows targeted elimination of pathogenic bacteria while preserving beneficial microbiota, reducing the collateral damage associated with broad-spectrum antibiotics (Gorski et al., 2020; Gabuat et al., 2025). Phage-encoded depolymerases are not universal to all therapeutic phages but are found in specific phage subsets that have evolved to target particular exopolysaccharide structures characteristic of specific bacterial species. The efficacy of depolymerase-mediated biofilm disruption is therefore dependent on matching the appropriate phage-encoded enzyme to the target pathogen’s biofilm matrix composition, underscoring the need for careful phage characterization prior to therapeutic use (Chen et al., 2024). Additionally, phages exhibit self-amplification at infection sites, with their numbers increasing in proportion to bacterial density and declining as the bacterial population is eliminated (Gabuat et al., 2025). These properties, combined with their ability to evolve alongside bacterial hosts, position phages as adaptable weapons against MDR pathogens.

### 1.3 Historical context and modern revival

While phage therapy was pioneered in the early 20th century, particularly in Eastern Europe and the former Soviet Union, it was largely abandoned in Western medicine following the advent of antibiotics. The current antimicrobial resistance crisis has catalyzed a resurgence of interest, with substantial increases in clinical applications, establishment of specialized phage therapy centers, and growing regulatory frameworks to support clinical development (Pirnay et al., 2021). This mini review synthesizes recent evidence on phage therapy against MDR bacteria, focusing on developments from 2020 to 2026 that have advanced the field toward mainstream clinical adoption.

## 2 Recent developments in phage therapy (2020–2026)

### 2.1 Expansion of clinical experience

Recent years have witnessed a significant expansion of specialized phage therapy centers and compassionate-use programs worldwide. The establishment of dedicated facilities with Good Manufacturing Practice (GMP)-compliant production capabilities has enabled systematic clinical application of phage therapy for patients with refractory MDR infections (Aslam et al., 2020; Pirnay et al., 2021). These centers have accumulated substantial clinical experience, demonstrating the feasibility and safety of intravenous and topical phage administration in patients who failed conventional antibiotic therapy.

The IPATH (Intravenous Phage Therapy) program at the University of California San Diego has provided critical insights into personalized phage therapy for severe MDR infections. A published series of the first 10 consecutive compassionate-use cases achieved a 70% clinical success rate (7/10 patients) with no major adverse events, illustrating the feasibility of personalized phage matching and the importance of iterative phage selection (Aslam et al., 2020). An expanded access cohort of 12 customized phage cases demonstrated 42% bacterial eradication and 66% overall favorable clinical response, with *in vitro* phage-antibiotic synergy documented in the majority of cases.

Infection types treated in the IPATH program included: - Pulmonary infections, particularly cystic fibrosis-related *Pseudomonas aeruginosa* infections treated with personalized nebulized phage therapy - Bloodstream infections and bacteremia, including recurrent septicemia in vascular device-associated infections - Device-related infections and osteomyelitis, representing the majority of reported cases - Intra-abdominal infections, including complex disseminated cases - Urinary tract infections, including ESBL *Escherichia coli* in post-transplant patients.

Pathogens targeted included: - *Pseudomonas aeruginosa* (most commonly reported) - Extended-spectrum beta-lactamase (ESBL)-producing *Escherichia coli* - *Acinetobacter baumannii* - *Stenotrophomonas maltophilia* - Vancomycin-resistant *Enterococcus faecium* (VRE).

Importantly, phages were almost universally administered in combination with antibiotics rather than as monotherapy in published IPATH-associated reports. Intravenous, topical/local, intra-abdominal, and nebulized routes were employed depending on infection site. This combination paradigm reflects the synergistic approach where phages are used to restore antibiotic susceptibility or provide complementary bactericidal activity against otherwise treatment-refractory MDR pathogens (Aslam et al., 2020).

Similar programs in Europe and other regions have reported comparable safety profiles and encouraging efficacy signals in compassionate-use settings (Pirnay et al., 2021).

### 2.2 Regulatory progress and clinical trials

Significant progress has been made in developing regulatory pathways for phage therapy in Western medicine. National centers and investigators have completed GMP-aligned clinical trials, with additional studies underway (Pirnay et al., 2021). While large randomized controlled trials remain limited, the regulatory landscape is evolving to accommodate the unique characteristics of phage therapy, including personalized phage selection and adaptive treatment protocols (Gorski et al., 2020; Pirnay et al., 2021).

### 2.3 Target pathogens and clinical indications

Clinical and preclinical work has emphasized problematic MDR pathogens including *Pseudomonas aeruginosa*, *Klebsiella pneumoniae*, *Acinetobacter baumannii*, and *Staphylococcus aureus* (Chen et al., 2024; Elfadadny et al., 2025; Tarasenko et al., 2025; Wang et al., 2025; Zhao et al., 2026). Multiple case reports and small studies have documented resolution of severe infections when standard therapies failed, including respiratory tract infections, wound infections, osteomyelitis, and bacteremia (Iszatt et al., 2021; Munshi et al., 2025). The clinical experience has been particularly notable for biofilm-associated infections and chronic wounds, where conventional antibiotics often fail to achieve bacterial eradication (Chen et al., 2024).

## 3 Engineered phage therapeutics: from lytic enhancement to non-replicative delivery platforms

### 3.1 Genetic engineering approaches

Advances in synthetic biology and genome engineering have revolutionized the development of therapeutic phages. Modern engineering approaches include homologous recombination-based editing, recombineering methods (BRED, BRIP), CRISPR-assisted genome alteration, chemical evolution, and genome rebooting to enhance lysis, expand host range, and deliver antimicrobial payloads (Khambhati et al., 2022; Peng et al., 2024). These techniques enable precise modification of phage genomes to improve therapeutic properties while maintaining or enhancing lytic activity.

Recombineering methods allow rapid generation of defined mutants for therapy through precise genome edits in phage genomes (Peng et al., 2024). CRISPR-Cas assisted editing enables targeted gene insertion or deletion and permits phages to carry antimicrobials or disable bacterial resistance genes (Khambhati et al., 2022). Chemically accelerated evolution (CAVE) expands host range through broadband phenotypic adaptation without requiring defined edits (Peng et al., 2024). Genome rebooting and synthetic assembly permit construction of minimal structural particles or bespoke payloads through *in vitro* assembly of modular phage genomes (Peng et al., 2024).

### 3.2 CRISPR-based phage therapeutics

A particularly promising development is the use of phages as delivery vehicles for CRISPR-Cas antimicrobial systems. Engineered Phage Inducible Chromosomal Islands (ePICIs) and related constructs deliver CRISPR-Cas modules that mediate bactericidal activity without chromosomal integration, broadening host targeting and reducing replication-associated risks in localized infections (Khambhati et al., 2022; Garmendia-Antolín et al., 2026). These systems have demonstrated efficacy comparable to antibiotics in animal models of localized infection, highlighting the potential of non-replicative therapeutic options.

Recent work has demonstrated targeted elimination of *Staphylococcus aureus* mastitis infections using synthetic phage-based CRISPR-Cas delivery systems (Garmendia-Antolín et al., 2026). These engineered systems combine the targeting specificity of phages with the programmable antimicrobial activity of CRISPR-Cas, offering a powerful approach to combat MDR pathogens while minimizing off-target effects.

As of 2026, CRISPR-based phage therapeutics have entered early-phase human clinical trials but have not received regulatory approval for routine clinical use. Two products have advanced to Phase I/II human studies:

1. LBP-EC01 (Locus Biosciences): A CRISPR-Cas3-enhanced bacteriophage cocktail targeting *E. coli* has completed Phase I/II evaluation for uncomplicated urinary tract infections, demonstrating favorable safety, pharmacokinetics, and pharmacodynamic profiles in human subjects.
2. SNIPR001 (SNIPR Biome): Completed a Phase I randomized, double-blind, first-in-human, dose-escalation study.

While these early-phase results are encouraging, no CRISPR-based phage therapeutic has received marketing authorization from the FDA, EMA, or equivalent regulatory bodies as of 2026. These platforms remain investigational and are accessible only through registered clinical trials or, in exceptional circumstances, compassionate use protocols.

### 3.3 Non-replicative phage-based delivery systems

Synthetic assemblies of phage structural components have been developed as non-replicative delivery vehicles for antibiotics and antimicrobial payloads. Cell-free assembled T7 structural particles loaded with penicillin reduced penicillin-resistant *Escherichia coli* populations in preclinical assays, demonstrating delivery of existing drugs via phage architecture (Shim, 2024). This approach represents a novel paradigm that leverages phage targeting capabilities without requiring phage replication, potentially addressing safety concerns associated with replicating phages.

Engineered ePICIs delivering CRISPR-Cas9 target bacteria with lethal precision and have shown efficacy comparable to antibiotics in animal models of localized infection (Garmendia-Antolín et al., 2026). These non-replicative systems offer advantages for infections where phage replication may be limited or where concerns about horizontal gene transfer are paramount. The programmable nature of CRISPR-Cas systems allows rapid adaptation to emerging resistance mechanisms and targeting of specific virulence or resistance genes (Khambhati et al., 2022; Garmendia-Antolín et al., 2026).

### 3.4 Comparative analysis: lytic phages vs. synthetic phage-based delivery platforms

The biological and clinical implications between naturally lytic phages and synthetic phage-based delivery platforms differ substantially:

**Table:** 

Feature	Naturally Lytic Phages	Synthetic Phage-Based Delivery Platforms
**Replication**	Self-replicating *in vivo*	Non-replicative; fixed dose
**Mechanism**	Infect, replicate, lyse bacteria	Deliver antimicrobial cargo without replication
**Resistance risk**	High (phage-resistant mutants can emerge)	Lower (no replication-driven selection pressure)
**Host range**	Narrow; requires precise matching	Can be engineered for broader targeting
**Horizontal gene transfer (HGT) risk**	Present (via transduction)	Reduced (non-replicative design minimizes transfer)
**Dosing**	Potentially self-amplifying at infection site	Requires sufficient initial dose; no amplification
**Regulatory classification**	Biological product (replicating organism)	Gene therapy/drug delivery vehicle
**Clinical development stage**	Compassionate use; early trials	Phase I/II (LBP-EC01, SNIPR001)
**Manufacturing complexity**	Requires bacterial host propagation	Cell-free assembly possible
**Biofilm penetration**	Dependent on depolymerase presence	Dependent on cargo and targeting moiety

This distinction is clinically important because the two approaches differ in their safety profiles, regulatory pathways, manufacturing requirements, and appropriate clinical indications. Naturally lytic phages may be preferred for infections where self-amplification is advantageous and where narrow host specificity is desired to preserve microbiota. Non-replicative platforms may be preferred where biosafety concerns are paramount, where broader targeting is needed, or where delivery of specific antimicrobial payloads (antibiotics, CRISPR systems) is the primary goal.

### 3.5 Biosafety considerations for engineered phage therapeutics

While engineered phage therapeutics offer substantial advantages, a balanced discussion must include explicit treatment of biosafety concerns:

**1. Horizontal Gene Transfer (HGT) Risks:** Engineered genetic elements carried by phage vectors may transfer to bacterial hosts or environmental microorganisms through transduction, conjugation, or plasmid mobilization. This risk is particularly relevant when phages carry CRISPR-Cas cassettes or antibiotic resistance gene-disrupting constructs. Mitigation strategies include use of non-replicative phage particles, incorporation of genetic containment sequences (e.g., kill-switch circuits), and engineering of narrow host ranges to limit unintended bacterial targets.
**2. Off-Target CRISPR Effects:** CRISPR guide RNAs can produce unintended cleavage at genomic sites with partial sequence complementarity, potentially affecting non-target bacteria including commensal microorganisms. In complex microbial communities (e.g., gut microbiome), the ecological consequences of off-target activity are difficult to predict. Mitigation requires comprehensive bioinformatic screening of guide RNA specificity, experimental validation in diverse microbial backgrounds, and consideration of high-fidelity Cas variants with reduced off-target activity.
**3. Immunogenicity:** Host immune responses to phage structural proteins and CRISPR components can neutralize therapeutic activity and raise safety concerns for repeated dosing. Neutralizing antibodies may develop following repeated phage administration, potentially limiting efficacy of subsequent treatments with the same phage (Aslam et al., 2020; Fattahi and Akhavan-Niaki, 2026). Strategies to address immunogenicity include PEGylation of phage particles, use of immunologically distinct phage cocktails for sequential treatments, and co-administration of immunomodulatory agents.
**4. Ecological Impacts:** Release of engineered phages may perturb microbiome composition and select for phage-resistant or CRISPR-resistant bacterial variants. The long-term ecological consequences of introducing engineered phages into human microbiomes and environmental reservoirs require careful monitoring and risk assessment.
**5. Resistance Evolution:** Phage-resistant mutants can emerge through receptor modification, restriction-modification systems, or CRISPR-Cas bacterial defense mechanisms, potentially reducing long-term efficacy. Combination strategies (phage cocktails, phage-antibiotic combinations) and iterative phage selection can help manage resistance evolution.
**6. Regulatory and Manufacturing Requirements:** GMP-grade production, standardized safety testing, and robust regulatory frameworks are essential prerequisites for clinical translation. These biosafety considerations do not preclude clinical development but necessitate rigorous preclinical characterization and ongoing pharmacovigilance.

## 4 Clinical applications and trials

### 4.1 Compassionate use programs

Compassionate-use programs have provided critical clinical experience and safety data for phage therapy. These programs typically involve patients with life-threatening MDR infections who have exhausted conventional treatment options. The accumulated experience demonstrates that phage therapy can be administered safely via multiple routes (intravenous, topical, inhalational, oral) and can achieve clinical success in a substantial proportion of cases (Aslam et al., 2020; Pattnaik et al., 2024). However, it is essential to recognize the significant limitations of this evidence base, as discussed in section “6.5. Clinical evidence gaps.”

### 4.2 Infection types and clinical outcomes

Phage therapy has been applied to diverse infection types, including respiratory tract infections, skin and soft tissue infections, bone and joint infections, urinary tract infections, and bloodstream infections (Iszatt et al., 2021; Pattnaik et al., 2024; Elfadadny et al., 2025; Munshi et al., 2025). Preliminary evidence suggests encouraging outcomes for biofilm-associated infections, where phage-encoded depolymerases can disrupt extracellular polymeric substances and improve bacterial clearance (Chen et al., 2024).

For respiratory tract infections caused by MDR pathogens, early-stage data indicate promise in both cystic fibrosis patients with chronic *Pseudomonas aeruginosa* infections and patients with ventilator-associated pneumonia (Iszatt et al., 2021; Elfadadny et al., 2025). Topical phage therapy has shown preliminary efficacy in chronic wound infections and diabetic foot ulcers, where biofilm formation and poor antibiotic penetration limit conventional treatment success (Pattnaik et al., 2024).

### 4.3 Personalized phage selection

A key feature of modern phage therapy is personalized phage selection based on *in vitro* susceptibility testing of patient isolates. This approach ensures optimal phage-bacteria matching and allows for iterative adjustment of phage cocktails if bacterial resistance emerges during treatment (Aslam et al., 2020). Specialized phage therapy centers maintain extensive phage libraries and have developed rapid screening protocols to identify effective phages for individual patients (Aslam et al., 2020; Pirnay et al., 2021).

## 5 Mechanisms and combination therapies

### 5.1 Mechanisms of phage-mediated bacterial killing

Phages kill bacteria through a multi-step process involving adsorption to surface receptors, genome injection, intracellular replication, and host lysis mediated by phage-encoded endolysins and holins (Gorski et al., 2020; Gabuat et al., 2025). The specificity of phage-receptor interactions provides the basis for targeted bacterial killing while sparing commensal microbiota. Phage-encoded depolymerases, when present in specific phage subsets, can enzymatically degrade biofilm matrices, improving penetration and killing of sessile bacterial communities where antibiotics often fail (Gorski et al., 2020; Chen et al., 2024).

### 5.2 Phage-antibiotic synergy

Multiple studies have documented additive or synergistic interactions between phages and antibiotics. Phage cocktails combined with antibiotics have shown improved outcomes in animal models and clinical case series for MDR pathogens (Chen et al., 2024; Rahimian et al., 2024). The mechanisms underlying synergy are diverse and may include phage-mediated disruption of biofilms enhancing antibiotic penetration, phage-induced metabolic changes increasing antibiotic susceptibility, and complementary targeting of different bacterial subpopulations (Rahimian et al., 2024).

### 5.3 Combination therapy strategies

Strategic approaches to combination therapy include sequential administration to limit resistance development, cocktails targeting multiple bacterial receptors, and engineered phages delivering enzymes or sensitizers to restore antibiotic efficacy (Peng et al., 2024; Rahimian et al., 2024). Clinical experience suggests that combination regimens may be particularly valuable for severe infections, biofilm-associated infections, and situations where rapid bacterial clearance is critical (Chen et al., 2024; Rahimian et al., 2024).

## 6 Challenges and limitations

### 6.1 Bacterial resistance to phages

Bacteria can evolve resistance to phages through receptor mutations, production of extracellular matrix that blocks phage access, or activation of defense systems such as CRISPR-Cas or restriction-modification systems (Peng et al., 2024). Phage resistance can emerge during therapy and may limit treatment efficacy. Countermeasures include rotating phage cocktails, engineering phages with broadened host range, and combining phages with antibiotics to suppress resistance evolution (Peng et al., 2024; Rahimian et al., 2024).

### 6.2 Host immune interactions

Innate and adaptive immune responses can neutralize phages or alter their pharmacokinetics. Neutralizing antibodies may develop following repeated phage administration, potentially limiting efficacy of subsequent treatments with the same phage (Aslam et al., 2020; Fattahi and Akhavan-Niaki, 2026). However, evidence also suggests that phages may exert immunomodulatory effects that contribute to therapeutic efficacy, though the mechanisms require further clarification in humans (Gorski et al., 2020; Fattahi and Akhavan-Niaki, 2026).

### 6.3 Manufacturing and quality control

Good Manufacturing Practice production of therapeutic phages presents unique challenges related to phage propagation, purification, quality control, and standardized potency assays (Pirnay et al., 2021). Ensuring consistent phage titers, purity, and absence of endotoxins and bacterial contaminants requires specialized expertise and infrastructure. The development of scalable manufacturing processes that can support both personalized therapy and off-the-shelf phage products remains a priority (Pirnay et al., 2021; Pattnaik et al., 2024).

### 6.4 Regulatory pathways

The regulatory landscape for phage therapy is evolving but remains heterogeneous across jurisdictions. Key challenges include defining appropriate quality attributes for phage products, establishing clinical trial designs that accommodate personalized phage selection, and developing expedited pathways for compassionate use linked to systematic data collection (Pirnay et al., 2021). Harmonization of regulatory approaches internationally would facilitate clinical development and broader access to phage therapy.

### 6.5 Clinical evidence gaps

While the clinical cases and small series reported between 2020 and 2026 provide encouraging preliminary signals of safety and potential efficacy, it is essential to recognize the significant limitations of the current evidence base. The overwhelming majority of published clinical outcomes derive from compassionate-use cases, uncontrolled case series, and retrospective analyses—study designs that are prone to reporting bias, lack standardized endpoints, and cannot establish causal efficacy (Gorski et al., 2020; Pirnay et al., 2021).

The absence of adequately powered, randomized, placebo-controlled trials means that definitive conclusions regarding clinical efficacy, optimal dosing regimens, treatment duration, patient selection criteria, and long-term safety cannot yet be drawn. Most clinical evidence comprises case series and compassionate-use reports, creating uncertainty about optimal indications, dosing regimens, treatment duration, and clinical endpoints (Gorski et al., 2020; Pirnay et al., 2021).

The field requires substantial investment in rigorous clinical trial infrastructure before phage therapy can transition from compassionate-use exception to evidence-based standard of care. Well-designed clinical trials are needed to define the role of phage therapy in the antimicrobial armamentarium and to support regulatory approval and reimbursement decisions.

## 7 Future perspectives

### 7.1 Integration into clinical practice

The integration of phage therapy into mainstream clinical practice will require continued progress in multiple domains. Standardized clinical pipelines must be developed, including curated phage libraries, rapid susceptibility screening protocols, and trial-ready GMP production processes to enable timely personalized therapy (Pirnay et al., 2021; Pattnaik et al., 2024). Clinical decision algorithms that define appropriate indications, patient selection criteria, and treatment protocols will facilitate adoption by clinicians unfamiliar with phage therapy.

### 7.2 Combination with other antimicrobial strategies

Future therapeutic approaches are likely to involve rational combinations of phages with antibiotics, antimicrobial peptides, immunomodulators, and other agents. Understanding the pharmacodynamic interactions between phages and other antimicrobials will enable optimization of combination regimens for specific pathogens and infection types (Rahimian et al., 2024; Zhao et al., 2026). The development of standardized combination protocols based on mechanistic understanding and clinical evidence will be important for maximizing therapeutic efficacy while minimizing resistance development.

### 7.3 Precision medicine approaches

Advances in rapid diagnostics, genomic sequencing, and computational prediction will enable increasingly precise matching of phages to individual patients and pathogens. Integration of phage susceptibility testing into clinical microbiology workflows, coupled with genomic surveillance of pathogens, will support real-time treatment optimization and resistance monitoring (Pattnaik et al., 2024; Peng et al., 2024). The development of point-of-care diagnostics that can rapidly identify suitable phages for a given infection would greatly expand the accessibility of phage therapy.

### 7.4 Regulatory innovation

Continued regulatory innovation is needed to accommodate the unique characteristics of phage therapy while ensuring patient safety and treatment efficacy. Adaptive regulatory frameworks that allow for personalized phage selection, iterative treatment optimization, and expedited compassionate use pathways linked to systematic data collection will be critical (Pirnay et al., 2021). International harmonization of regulatory approaches would facilitate clinical development and enable broader access to phage therapy globally.

### 7.5 Manufacturing scale-up

Scalable manufacturing processes that can support both personalized therapy and off-the-shelf phage products will be essential for widespread clinical adoption. Advances in bioreactor design, purification technologies, and quality control methods are needed to enable cost-effective production of therapeutic phages at clinical scale (Pirnay et al., 2021; Pattnaik et al., 2024). The development of stable formulations with extended shelf life would improve the practicality of maintaining diverse phage libraries in clinical settings.

## 8 Conclusion

Phage therapy has re-emerged as a promising approach to combat MDR bacterial infections, supported by expanding clinical experience, sophisticated engineering capabilities, and growing mechanistic understanding. Recent developments from 2020 to 2026 demonstrate significant progress in clinical applications, with specialized phage therapy centers accumulating safety data and preliminary efficacy signals through compassionate-use programs and early clinical trials. Engineering advances, particularly CRISPR-based delivery systems and synthetic phage particles, have expanded therapeutic possibilities beyond traditional lytic phages.

However, it is essential to acknowledge that the current evidence base remains limited. The overwhelming majority of clinical data derives from compassionate-use cases and uncontrolled case series, which cannot establish definitive efficacy. Large randomized controlled trials are urgently needed to validate the therapeutic potential of phage therapy and to define optimal clinical protocols.

While challenges remain in regulatory standardization, scalable manufacturing, immune interactions, and predictable resistance management, the field has demonstrated clear momentum toward clinical translation. The convergence of synthetic biology, personalized medicine approaches, and accumulating clinical evidence positions phage therapy as a viable complementary strategy in the antimicrobial armamentarium. Continued investment in clinical trials, manufacturing infrastructure, and regulatory innovation will be essential to realize the full potential of phage therapy in addressing the global crisis of antimicrobial resistance.

The path forward requires coordinated efforts across multiple domains: rigorous clinical trials to define optimal indications and protocols, continued engineering innovation to enhance therapeutic capabilities, development of scalable manufacturing processes, and evolution of regulatory frameworks to accommodate personalized biologics. With sustained progress in these areas, phage therapy has the potential to become an integral component of modern antimicrobial therapy, offering hope for patients with infections that have exhausted conventional treatment options.

## References

[B1] Aslam S. Lampley E. Wooten D. Karris M. Benson C. Strathdee S. et al. (2020). Lessons learned from the first 10 consecutive cases of intravenous bacteriophage therapy to treat multidrug-resistant bacterial infections at a single center in the United States. *Open Forum Infect. Dis.* 7:ofaa389. 10.1093/OFID/OFAA389 33005701 PMC7519779

[B2] Chen Y. Li X. Wang S. Guan J. Li Y. Sheng Y. et al. (2024). The application value of bacteriophage in patients with severe drug-resistant bacterial infections. *J. Transl. Crit. Care Med.* 6:100022. 10.1097/jtccm-d-24-00022

[B3] Elfadadny A. Ragab R. F. Abd Alaziz O. A. Essa H. Y. Eltony E. M. Ammar M. M. et al. (2025). Bacteriophage therapy in clinical practice: case studies of *Pseudomonas aeruginosa* infections. *J. Chemother.* [Online ahead of print]. 10.1080/1120009x.2025.2547147. 40810649

[B4] Fattahi S. Akhavan-Niaki H. (2026). Next-generation gene therapy for infectious disease: advances, challenges, and future directions. *J. Infect. Public Health* 19:103164. 10.1016/j.jiph.2026.103164 41671593

[B5] Gabuat J. C. D. Cayabyab M. K. T. Tiu C. J. D. (2025). Phage therapy for management of multi-drug resistance: unleashing nature’s tiny warriors to combat bacterial infections. *Acta Microbiol. Bulgarica* 41 203–215. 10.59393/amb25410203

[B6] Garmendia-Antolín I. Alonso-Lerma B. Barriales D. Reviriego I. Esteban I. Quesada-Ganuza A. et al. (2026). Targeted elimination of *Staphylococcus aureus* mastitis infections with synthetic phage-based CRISPR-Cas delivery systems. *NPJ Biofilms Microbiomes* 12:931. 10.1038/s41522-026-00931-X 41680202 PMC13013576

[B7] Gorski A. Miedzybrodzki R. Wegrzyn G. Jonczyk-Matysiak E. Borysowski J. Weber-Dabrowska B. (2020). Phage therapy: current status and perspectives. *Med. Res. Rev.* 40 459–463. 10.1002/med.21593 31062882

[B8] Hatfull G. F. Dedrick R. M. Schooley R. T. (2022). Phage therapy for antibiotic-resistant bacterial infections. *Annu. Rev. Med.* 73 197–211. 10.1146/ANNUREV-MED-080219-122208 34428079

[B9] Iszatt J. J. Larcombe A. N. Stick S. M. Kicic A. (2021). Phage therapy for multi-drug resistant respiratory tract infections. *Viruses* 13:1809. 10.3390/V13091809 34578390 PMC8472870

[B10] Khambhati K. Bhattacharjee G. Gohil N. Dhanoa G. K. Sagona A. P. Singh V. (2022). Phage engineering and phage-assisted CRISPR-Cas delivery to combat multidrug-resistant pathogens. *Bioeng. Transl. Med.* 7:e10381. 10.1002/btm2.10381 36925687 PMC10013820

[B11] Munshi R. Bashir S. M. Ahmad S. M. (2025). Phage therapy: navigating the mechanisms, benefits, and challenges in the fight against multidrug-resistant infections. *medRxiv* [Preprint]. 10.1101/2025.04.19.25325436

[B12] Pattnaik S. Priyadarshini P. Swain S. S. Sahu P. K. (2024). Bacteriophage as a potential biotherapeutics to combat present-day crisis of multi-drug resistant pathogens. *Heliyon* 10:e37489. 10.1016/j.heliyon.2024.e37489 39309956 PMC11416503

[B13] Peng H. Borg R. E. Dow L. P. Pruitt B. L. Chen I. A. (2024). Engineering phages to fight multidrug-resistant bacteria. *Chem. Rev.* 124 13040–13108. 10.1021/acs.chemrev.4c00681 39680919 PMC11758799

[B14] Pirnay J. P. Verbeken G. Ceyssens P. J. Huys I. De Vos D. Ameloot C. et al. (2021). Recent progress towards the implementation of phage therapy in Western medicine. *FEMS Microbiol. Rev.* 45:fuab040. 10.1093/FEMSRE/FUAB040 34289033

[B15] Rahimian K. Soleimani-Delfan A. Etemadifar Z. Emtiazi G. Khoshbakht R. (2024). A new insight into phage combination therapeutic approaches against drug-resistant mixed bacterial infections. *Phage* 5 67–79. 10.1089/phage.2024.0011 40045937 PMC11876824

[B16] Shim H. (2024). *Self-Assembling T7 Phage Syringes with Modular Genomes for Targeted Delivery of Penicillin Against Beta-Lactam-Resistant Escherichia coli.* Irvine: University of California. 10.1186/s12896-025-01003-2 PMC12220360 40597155

[B17] Tarasenko A. Krylov V. Pleteneva E. (2025). From crisis to cure: phage therapy’s clinical role in combating multidrug-resistant *Acinetobacter baumannii*. *OSF [Preprints].* 10.31219/osf.io/q53fm

[B18] Wang Y. Chen L. Bian X. Lu Y. Rao X. Hu F. (2025). Phage therapy as a revitalized weapon for treating clinical diseases. *Microbiome Res. Rep.* 4:31. 10.20517/mrr.2025.31 41133098 PMC12540056

[B19] Zhao M. Rong D. Chen L. Cai S. Lu Y. Dai J. et al. (2026). Beyond antibiotics: therapeutic strategies utilizing probiotics and bacteriophages against drug-resistant *Staphylococcus aureus*. *Microorganisms* 14:344. 10.3390/microorganisms14020344 41753631 PMC12943351

